# The Complete Structure of the Core Oligosaccharide from *Edwardsiella tarda* EIB 202 Lipopolysaccharide

**DOI:** 10.3390/ijms18061163

**Published:** 2017-05-31

**Authors:** Marta Kaszowska, Elena de Mendoza-Barberá, Anna Maciejewska, Susana Merino, Czeslaw Lugowski, Juan M. Tomás

**Affiliations:** 1 Department of Immunochemistry, Hirszfeld Institute of Immunology and Experimental Therapy, Polish Academy of Sciences, R. Weigla 12, PL-53-114 Wroclaw, Poland; aniaaugustyniuk@iitd.pan.wroc.pl (A.M.); lugowski@iitd.pan.wroc.pl (C.L.); 2 Department of Microbiology, University of Barcelona, Diagonal 643, 08071 Barcelona, Spain; edemendoza@ub.edu (E.d.M.-B.); smerino@ub.edu (S.M.); jtomas@ub.edu (J.M.T.); 3 Department of Biotechnology and Molecular Biology, University of Opole, PL-45-035 Opole, Poland

**Keywords:** *Edwardsiella tarda*, core oligosaccharide, MALDI-TOF MS, ESI MS^n^, NMR, genomic

## Abstract

The chemical structure and genomics of the lipopolysaccharide (LPS) core oligosaccharide of pathogenic *Edwardsiella tarda* strain EIB 202 were studied for the first time. The complete gene assignment for all LPS core biosynthesis gene functions was acquired. The complete structure of core oligosaccharide was investigated by ^1^H and ^13^C nuclear magnetic resonance (NMR) spectroscopy, electrospray ionization mass spectrometry MS^n^, and matrix-assisted laser-desorption/ionization time-of-flight mass spectrometry. The following structure of the undecasaccharide was established:

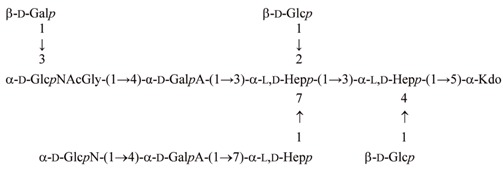

The heterogeneous appearance of the core oligosaccharide structure was due to the partial lack of β-d-Gal*p* and the replacement of α-d-Glc*p*NAcGly by α-d-Glc*p*NGly. The glycine location was identified by mass spectrometry.

## 1. Introduction

*Edwardsiella tarda* is a Gram-negative bacterium and a pathogen of farmed fish. It is the etiological agent of a systematic disease called edwardsiellosis, which has been reported to affect a wide range of freshwater and marine fish [1,2]. In addition to fish, *E. tarda* is also an occasional human pathogen and known to cause both gastroenteritis and extraintestinal infections in humans [3,4]. A number of virulence-associated systems and factors, such as the type III and type VI secretion systems, the LuxS/AI-2 quorum sensing system, and hemolysin systems, have been identified in *E. tarda* [5]. Additionally, a sialidase shows a potential pathogenicity and immunogenicity [6].

In Gram-negative bacteria, the lipopolysaccharide (LPS) is one of the major structural and immunodominant molecules of the outer membrane. It consists of three moieties: lipid A, core oligosaccharide, and *O*-specific polysaccharide (*O*-antigen). The *O*-antigen is the external component of LPS, and its structure consists of different number of repeating units. The *O*-specific polysaccharide chains are transferred to lipid A-core to form LPS, in a step involving WaaL, the putative bifunctional enzyme named *O*-antigen ligase. Another interesting feature is the high chemical variability shown by the *O*-antigen LPS, leading to a similar genetic variation in the genes involved in their biosynthesis, the so-called *wb* cluster (for a review, see [7]). Despite the emerging importance of this pathogenic microorganism, until now only four LPS structures of *E. tarda* strains were investigated [8,9,10,11].

In studies of several *Enterobacteriaceae* such as *Escherichia coli*, *Salmonella enterica*, and *Klebsiella pneumoniae*, genes involved in LPS core biosynthesis are usually found clustered in a region of the chromosome, the *waa* gene cluster [12,13]. On the other hand, a careful analysis of several full sequenced genomes suggested that genes for the LPS core biosynthesis may not be clustered and may be distributed between several regions, e.g., as in *Yersinia pestis* [14] or *Proteus mirabilis* [15]. In other cases, only a single gene involved in LPS core biosynthesis is out of the *waa* gene cluster, for instance, *Plesiomonas shigelloides* [16]. Nothing is known about the genomics or the LPS core structure from any *E. tarda* strain, besides the role played by the *waaL* (*O*-antigen ligase) characterized from strain EIB 202 [17]. *E. tarda* strain EIB 202 was isolated from moribund fish *Scophthalmus maximum* in a marine culture farm in China [18], and its full genome sequenced [19].

Here, the chemical structure of the core oligosaccharide in a pathogenic strain of *E. tarda* EIB 202 to proceed with the genomics of the core biosynthesis is reported for the first time.

## 2. Results

### 2.1. Isolation of the Core Oligosaccharide

LPS of *E. tarda* EIB 202 was isolated from bacterial mass with a yield of 0.5%. The mild acid hydrolysis of the LPS yielded eight polysaccharide (PS) and oligosaccharide (OS) fractions: PSI-VI consisting of a core oligosaccharide substituted by several repeating units, and OSVII and OSVIII—the unsubstituted core oligosaccharide fractions. The high yield of PSI-VI suggested the smooth (S-LPS) type of *E. tarda* EIB 202 LPS. The data presented herein concern the OSVIII fraction. The differences between OSVII and OSVIII fractions are presented herein based on MALDI-TOF MS (matrix assisted laser desorption/ionization-time of flight mass spectrometry) and ESI MS^n^ (electrospray ionization mass spectrometry) analysis.

### 2.2. Structure Analysis of the Core Oligosaccharide Fractions

The chemical analyses of OSVIII showed the presence of 2,3,7-trisubstituted l,d-Hep*p*, 3,4-disubstituted l,d-Hep*p*, 7-substituted l,d-Hep*p*, two terminal d-Glc*p*N, two terminal d-Glc*p*, two 4-substituted d-Gal*p*A, and 5-substituted Kdo*p*. The analyses of OSVII showed the presence of monosaccharides identified for OSVIII and additionally two sugar residues: the terminal d-Gal*p*, and 3-substituted d-Glc*p*NAc was identified instead of the terminal d-Glc*p*N in OSVIII.

**Figure 1:**
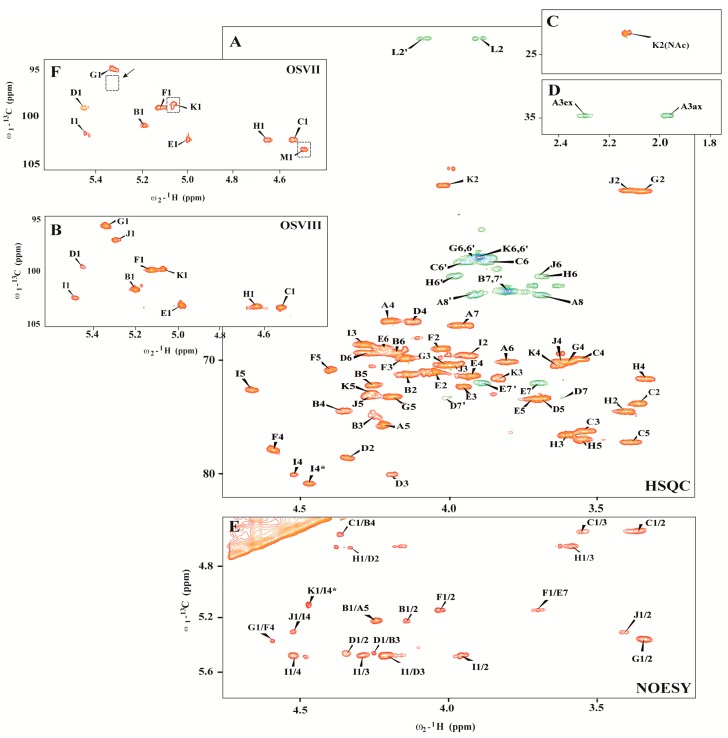
(**A**–**D**) Selected regions of the ^1^H–^13^C HSQC-DEPT (heteronuclear single-quantum correlation–distortionless enhancement by polarization transfer) and (**E**) ^1^H–^1^H NOESY (nuclear overhauser spectroscopy) spectra of the fraction OSVIII of *Edwardsiella tarda* EIB 202 lipopolysaccharide (LPS); (**F**) anomeric region of the ^1^H–^13^C HSQC-DEPT spectrum of the fraction OSVII with marked difference in comparison with OSVIII. The cross-peaks are labeled as shown in the text.

The ^1^H NMR (nuclear magnetic resonance) spectrum of the OSVIII contained main signals for nine anomeric protons, and signals characteristic for the deoxy protons of Kdo*p* residue belongs to part of the core oligosaccharide (residues **A**–**J**). The HSQC-DEPT (heteronuclear single-quantum correlation-distortionless enhancement by polarization transfer) spectra obtained for the OSVIII fraction contained signals for nine major anomeric protons and carbons, and Kdo spin systems, respectively (Figure 1 and Table 1).

Residue **A** was identified as the 5-substituted Kdo on the basis of characteristic deoxy proton signals at δ_H_ 1.96 ppm (H-3*ax*) and δ_H_ 2.29 ppm (H-3*eq*), as well as a high chemical shift of the C-5 signal (δ_C_ 75.7 ppm). Residue **B** (δ_H_/δ_C_ 5.19/101.7 ppm, ^1^*J*_C-1,H-1_ ~175 Hz) was recognized as the 3,4-disubstituted l-*glycero*-α-d-*manno*-Hep*p* residue on the basis of the small vicinal couplings between H-1, H-2, and H-3 and relatively high chemical shifts of the C-3 (δ_C_ 74.7 ppm) and the C-4 (δ_C_ 74.4 ppm) signals. Residue **C** (δ_H_/δ_C_ 4.51/103.3 ppm, ^1^*J*_C-1,H-1_ ~163 Hz) and residue **H** (δ_H_/δ_C_ 4.63/103.2 ppm, ^1^*J*_C-1,H-1_ ~166 Hz) were recognized as the β-d-Glc*p* on the basis of the large vicinal couplings between all protons in the sugar ring. Residue **D** (δ_H_/δ_C_ 5.44/99.6 ppm, ^1^*J*_C-1,H-1_ ~175 Hz) was recognized as the 2,3,7-trisubstituted l-*glycero*-α-d-*manno*-Hep*p* residue from the ^1^H and ^13^C chemical shift values, small vicinal couplings between H-1, H-2, and H-3 and relatively high chemical shift values of the C-2 (δ_C_ 78.6 ppm), C-3 (δ_C_ 80.0 ppm), and C-7 (δ_C_ 73.3 ppm) signals. Residue **E** (δ_H_/δ_C_ 4.98/103.2 ppm, ^1^*J*_C-1,H-1_ ~173 Hz) was recognized as the 7-substituted l-*glycero*-α-d-*manno*-Hep*p* from the ^1^H and ^13^C chemical shifts, the small vicinal couplings between H-1, H-2, and H-3 and the relatively high chemical shift value of the C-7 (δ_C_ 72.0 ppm) signal. Residues **F** (δ_H_/δ_C_ 5.12/99.8 ppm, ^1^*J*_C-1,H-1_ ~175 Hz) was recognized as the 4-substituted α-d-Gal*p*A based on the characteristic five proton spin system, the high chemical shifts of the H-3 (δ_H_ 4.16 ppm), H-4 (δ_H_ 4.62), H-5 (δ_H_ 4.41), and C-4 (δ_C_ 77.9 ppm) signals, the large vicinal couplings between H-2 and H-3 and small vicinal coupling between H-3, H-4, and H-5. Residue **I** (δ_H_/δ_C_ 5.47/102.5 ppm, ^1^*J*_C-1,H-1_ ~174 Hz) was also recognized as the 4-substituted α-d-Gal*p*A residue based on the similar characteristic five proton spin system. Residues **G** (δ_H_/δ_C_ 5.33/95.6 ppm, ^1^*J*_C-1,H-1_ ~176 Hz) and **J** (δ_H_/δ_C_ 5.29/97.0 ppm, ^1^*J*_C-1,H-1_ ~176 Hz) were recognized as the terminal α-d-Glc*p*N due to the large coupling between H-1, H-2, and H-3 and the small vicinal coupling between H-3, H-4, and H-5, as well as the chemical shift value of the C-2 (δ_C_ 55.1 and δ_C_ 55.1 for **G** and **J**, respectively). The 1D ^31^P NMR spectrum showed no indication of phosphate groups in the OSVIII.

Additionally, the residue **K** (δ_H_/δ_C_ 5.06/99.8 ppm, ^1^*J*_C-1,H-1_ ~165 Hz) was recognized as the terminal α-d-Glc*p*NAc from a low ^13^C chemical shift of the C-2 signal (δ_C_ 54.6 ppm), and the large vicinal couplings between all ring protons. The *N*-acetyl group at δ_H_/δ_C_ 2.13/23.2 ppm (δ_C_ 175.9 ppm) was identified. The presence of heterogeneity in OSVIII was due to partial replacement of α-d-Glc*p*N (residue **J**) by α-d-Glc*p*NAc (residue **K**). The last sugar residue **M** (δ_H_/δ_C_ 4.45/103.6 ppm, ^1^*J*_C-1,H-1_ ~163 Hz), identified only in OSVII, was recognized as the terminal β-d-Gal*p* due to the large vicinal couplings between H-1, H-2, and H-3 and the small vicinal couplings between H-3, H-4, and H-5. The terminal residue **M** in OSVII is linked to C-3 of → 3)-α-d-Glc*p*NAc (residue **K**) corresponding to the terminal form of this residue in the OSVIII.

In the HSQC-DEPT spectra of OSVIII (at δ_H_/δ_C_ 3.90/41.8 ppm), additional negative CH_2_ signals were detected. These resonances showed correlation with a carbonyl carbon signals at δ_C_ 168.0 ppm in the HMBC (heteronuclear multiple bond correlation) spectra, suggesting the presence of glycine (residue **L**). This residue was also confirmed by mass spectrometry.

The monosaccharide sequence in OSVIII was established using a NOESY (nuclear overhauser spectroscopy) and HMBC experiments. NOESY spectra showed strong inter-residue cross-peaks between the following transglycosidic protons: H-1 of **B**/H-5 of **A**, H-1 of **D**/H-3 of **B**, H-1 of **C**/H-4 of **B**, H-1of **E**/H-7,7′ of **D**, H-1 of **F**/H-7,7′ of **E**, H-1 of **G/**H-4 of **F**, H-1 of **H**/H-2 of **D**, H-1 of **I**/H-3 of **D**, and H-1 of **J**/H-4 of **I**. (Figure 1E). The HMBC spectrum of OSVIII confirmed substitution positions of all of the monosaccharide residues (data not shown). Additionally, NOESY showed the cross-peak between H-1 of **K** and H-4 of I*, and it also provided evidence for the heterogeneity of OSVIII with the presence of α-d-Glc*p*NAc and as a substitution of 4-substituted α-d-Gal*p*A (residue **I**) in OSVIII.

**Figure 2:**
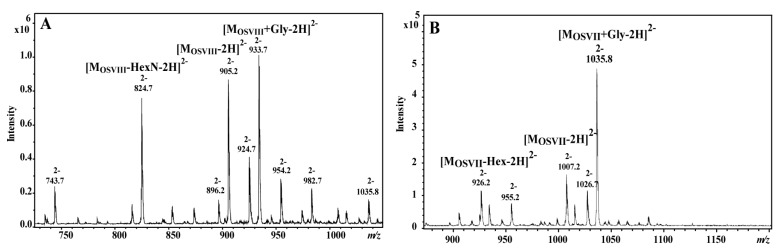
ESI (electrospray ionization) mass spectra of the core oligosaccharide fractions (**A**) OSVIII and (**B**) OSVII of *E. tarda* EIB 202.

The OSVIII and OSVII fractions were analyzed by ES MS^n^ and MALDI-TOF MS/MS. Ten sugar residues: two Glc, three Hep, two GalA, two GlcN, and Kdo, give together a monoisotopic mass of 1812.567 Da (M_OSVIII_). Eleven sugar residues from OSVII give together a monosotopic mass of 2016.630 Da. The negative ESI MS^n^ (Figure 2A) mass spectrum of OSVIII showed the main ion at *m*/*z* 905.2 [M_OSVIII_-2H]^2−^ correspond to core oligosaccharide (OSVIII), and ions correspond to core substituted with the glycine (Gly) residue at *m*/*z* 933.7 [M_OSVIII_+Gly-2H]^2−^ and *m*/*z* 924.7 [M_OSVIII_+Gly-H_2_O-2H]^2−^. The negative ESI MS^n^ mass spectrum of OSVII showed the main ion at *m*/*z* 1035.8 [M_OSVII_+Gly-2H]^2−^, which represented the complete core oligosaccharide substituted by Gly (Figure 2B).

**Figure 3:**
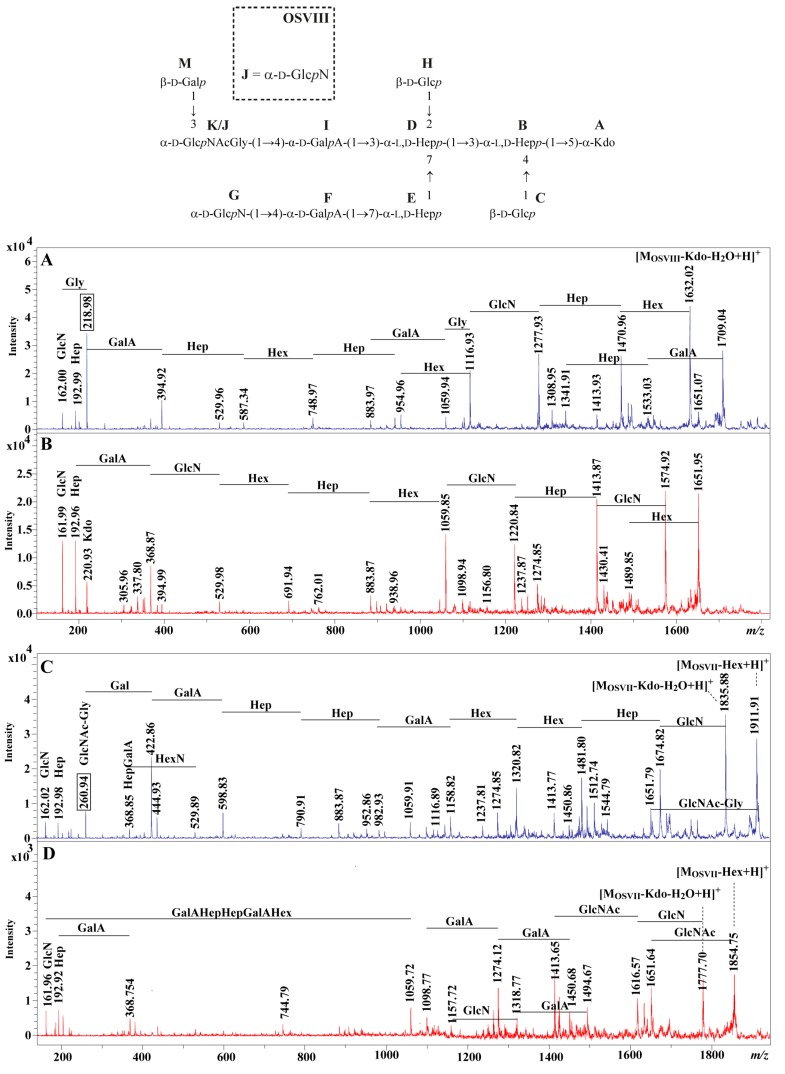
MALDI-TOF (matrix assisted laser desorption/ionization-time of flight) MS/MS fragmentation mass spectra of the ions (**A**) at *m*/*z* 1870.16 [M_OSVIII_+Gly+H]^+^, (**B**) at *m*/*z* 1813.15 [M_OSVIII_+H]^+^, (**C**) at *m*/*z* 2074.19 [M_OSVII_+Gly+H]^+^, and (**D**) at *m*/*z* 2017.19 [M_OSVII_+H]^+^ of *E. tarda* EIB 202, differing in the presence of glycine.

The location of glycine was determined by the positive ion mode MALDI-TOF MS/MS. The ions at *m*/*z* 1813.15 [M_OSVIII_+H]^+^, at *m*/*z* 1870.16 [M_OSVIII_+Gly+H]^+^, at *m*/*z* 2017.19 [M_OSVII_+H]^+^, and at *m*/*z* 2074.19 [M_OSVIII_+Gly+H]^+^ were selected for further fragmentations by the use of positive ion mode MALDI-TOF MS/MS. The main daughter ions detected in the MALDI-TOF MS/MS spectra were explained. In Figure 3A, the ion at *m*/*z* 162.00 corresponds to GlcN, while the ion at *m*/*z* 218.98 was explained by the GlcN–Gly. The similar pair of fragment ions at *m*/*z* 1116.93 and at *m*/*z* 1059.94 with the mass difference corresponding to the glycine residue was also identified. These ions were not identified on the spectrum of the ion *m*/*z* 1813.15 fragmentation (Figure 3B). In Figure 3C, the daughter ion at *m*/*z* 260.94 was subsequently attributed to the GlcNAc-Gly fragment. The similar pair of fragment ions at *m*/*z* 1911.91 and at *m*/*z* 1651.79 with the mass difference corresponding to the glycine residue was also identified. These ions were not identified on the spectrum of the ion at *m*/*z* 2017.19 fragmentation (Figure 3D). These observations indicate that glycine substitutes GlcN (residue J) in OSVIII and GlcNAc (residue K) in OSVII. The positions of glycine in OSVIII and OSVII were not determined.

### 2.3. Organization of the E. tarda Strain EIB 202 waa Gene Cluster

In most *Enterobacteriaceae* studied so far, the genes involved in core LPS biosynthesis were found clustered (*waa* gene cluster). When we inspected the currently available *E. tarda* strain EIB 202 genome, we found a clear region with the *waa* gene cluster (proteins encoded ETAE_0083 to ETAE_0072). This *waa* region, like in the majority of *Enterobacteriaceae*, is started by the *hldE* (encoded protein ETAE_0083), which codifies for the ADP-l-*glycero*-d-mannoheptose-6-epimerase, and the end flanked by the *coaD* (encoded protein ETAE_0071) codifying for phosphopantetheine adenylyltransferase [20].

Despite the genome annotation, it seems that more of the genes are shared by different *Enterobacteriaceae* mainly *K. pneumoniae* or *P. shigelloides*, which were previously characterized by us [7,14]. Table 2 shows proteins encoded from *E. tarda* EIB 202 *waa*.

## 3. Discussion

Here, the chemical structure and genomics of the complete undecasaccharide core structure of *E. tarda* EIB 202 LPS are presented for the first time. This core oligosaccharide is heterogeneous. The heterogeneity corresponded to the partial lack of β-d-Gal*p* and the replacement of α-d-Glc*p*NAcGly by α-d-Glc*p*NGly. The functions of the genes found in the *waa* gene cluster from the *E. tarda* strain EIB 202 seems to be in agreement with the chemical structure of the LPS-core. The *E. tarda* core LPS structure is highly similar to that of *K. pneumoniae* at least up to the outer-core residue GlcNI and *P. shigelloides* 302-73 in practically up to the last monosaccharide residue that links the *O*-antigen LPS [7,14]. WabH and WapB are enzymes that transfer GlcNAc to a GalA in different acceptor substrates of LPS-core in an α(1→4) linkage. WabG and WapC are enzymes that transfer GalA to a Hep also in different LPS-core substrates and with different linkage, α(1→3) and α(1→7), respectively. It is important to note that besides performing the same enzymatic functions the acceptor substrate differences determine that the enzymes showed very little homology; furthermore, WabG and WapC are more similar among them (26 identity and 47% similarity) than WabH and WapB are (24% identity and 46% similarity), except that the latter ones showed identical linkage. This point indicates the importance of the substrates in the enzymatic reactions to build-up the LPS-core molecules. *K. pneumoniae* WabK is a glycosyltransferase that incorporates a Glc residue in a β(1→4) to GlcN. *E. tarda waa* ORF4, according to the *E. tarda* strain EIB 202 LPS-core established, as well as their low homology but unique to WabK, could be the galatosyltransferase that incorporates a Gal residue in a β(1→4) to GlcNAc *E. tarda waa* Orf5 encoding for ETAE_0079 (Table 2). All genes from the *E. tarda waa* cluster were found in the *E. ictaluri* genomes, except for *wapB* and *wapC*, which seems to be unique for the species *E. tarda*. All of the other genes from the *E. tarda waa* cluster (*hldE*, *waaF*, *waaC*, *ETAE_0079*, *waaN*, *waaQ*, *wabG*, *wabH*, *waaA*, and *wapE*) show 98% or more identity to the related *E. ictaluri* LPS-core biosynthetic genes according to their genomes. Of course, *E. ictaluri waaL*, which is the *O*-antigen ligase, is a bifunctional enzyme recognizing the *O*-antigen LPS and the LPS-core, as it usual shows a reduced identity (56%) compared to *E. tarda waaL*. Nevertheless, the *E. ictaluri waaL*-encoded protein shows the typical transmembrane domains (data not shown).

The *E. tarda* LPS motif β-Glc-(1→2)-α-l-HepII seems not to be encoded by any of the glycosyltransferases found in the *waa* cluster. This LPS motif is identical to a previously studied by us in the *P. shigelloides* strain 302-73 encoded by WapG. For this reason, we decided to blastx the *P. shigelloides* 302-73 WapG [16] against the *E. tarda* strain EIB 202 genome. We found a clear unique candidate, the gene encoding ETAE_1955, which showed 58% identity and 74% similarity to WapG. We suggest that it could be responsible for β-Glc-residue linked to HepII-[(1→2)-α-l-HepII].

## 4. Materials and Methods

### 4.1. Growth Conditions and Isolation of the Lipopolysaccharide and the Polysaccharide

Bacteria *E. tarda* EIB 202 was obtained from the Y. Zhang laboratory [18]. The bacteria were grown and harvested as described previously [21]. The LPS was extracted from bacterial cells of *E. tarda* EIB 202 by the hot phenol/water method [22]. LPS (200 mg) was degraded by treatment with 1.5% acetic acid at 100 °C for 45 min. The supernatant was fractionated on a column (1.6 × 100 cm) of Bio-Gel P-10, equilibrated with 0.05 M pyridine/acetic acid buffer, pH 5.4. Eluates were monitored with a Knauer differential refractometer, and all fractions were checked by NMR spectroscopy and mass spectrometry (MALDI-TOF and ESI MS^n^).

### 4.2. Chemical Methods

Methylation of oligosaccharide fractions was performed according to the method described by Ciucanu and Kerek [23]. The absolute configurations of the monosaccharides were determined as described by Gerwig et al. [24]. Alditol acetates and partially methylated alditol acetates were analyzed by gas chromatography GC-MS with the Thermo Scientific TSQ system using an RX5 fused-silica capillary column (0.2 mm by 30 m) and a temperature program of 150 → 270 °C at 12 °C/min.

### 4.3. Instrumental Methods

All NMR spectra were recorded on a Bruker Avance III 600 MHz spectrometer equipped with a 5 mm QCI cryoprobe with z-gradient. The measurements were performed at 303 K without sample spinning and using the acetone signal (δ_H_/δ_C_ 2.225/31.05 ppm) as an internal reference. The signals were assigned by one- and two-dimensional experiments: ^1^H-^1^H COSY (correlation spectroscopy), TOCSY (total correlated spectroscopy), NOESY, ^1^H-^13^C HSQC-DEPT, HSQC-TOCSY, and HMBC. In the TOCSY experiments, the mixing times were 30, 60, and 100 ms. The NOESY experiment was performed with the mixing time of 200 ms, and HMBC experiment with a delay of 80 ms. For observation of phosphorus atoms, one-dimensional ^31^P NMR spectra were recorded. The data were acquired and processed using standard Bruker software. The processed spectra were assigned with the help of the SPARKY program [25].

Core oligosaccharide (1 mg/mL in mQ) fractions were analyzed using a MALDI-TOF Ultraflextreme (Bruker, Germany) instrument. The MALDI-TOF MS spectra were obtained in a positive ion mode. 2,5-Dihydroxybenzoic acid (10 mg/mL in 1:1 AcN/0.2 M citric acid [*v*/*v*]) was used as a matrix for analyses.

Negative-ion electrospray ionization mass spectra (ESI-MS^n^) were recorded using an Amazon SL (Bruker Daltonics, Bremen, Germany) ion trap mass spectrometer. The samples were dissolved in a 50 µg/mL acetonitrile-water-formic acid solution (50:50:0.5 [*v*/*v*/*v*]).

### 4.4. Comparative Genomics

For each analyzed genome we gathered all coding sequence (CDS) and pseudo-CDS information by parsing NCBI GenBank records. When we obtained the UniProt Knowledge Base records for these loci using the cross-reference with Entrez GeneIDs and parsed them for gene names, functional annotations, and associated COG, PFAM, and TIGRFAM protein domains were studied. To annotate orthologs, we wrote custom scripts to analyze reference sequence alignments made to subject genomes with blastn and tblastn via NCBI’s web application programming interface.

## Figures and Tables

**Table 1:** ^1^H and ^13^C NMR (nuclear magnetic resonance) chemical shifts of the core oligosaccharide of *E. tarda* EIB 212 LPS.

Residues	Oligosaccharide		Chemical Shifts (ppm)							
	OSVIII	OSVII	H1/C1	H2/C2	H3(ax,eq)/C3	H4/C4	H5/C5	H6,6`/C6	H7,7`/C7	H8,8`/C8 (NAc)
**A** →5)-Kdo	*	*	-	-	1.96, 2.29	4.21	4.25	3.81	3.97	3.69, 3.92
			nd	97.7	34.7	66.5	75.7	70.1	66.9	64.3
**B** →3,4)-l-*glycero*-α-d-*manno*-Hep*p*-(1→	*	*	5.19	4.15	4.25	4.35	4.26	4.18	3.80	
			101.7	71.3	74.7	74.4	72.2	69.4	63.9	
**C** →β-d-Glc*p*-(1→	*	*	4.51	3.36	3.55	3.56	3.55	3.87, 3.95		
			103.3	73.9	76.2	69.9	76.2	61.5		
**D** →2,3,7)-l-*glycero*-α-d-*manno*-Hep*p*-(1→	*	*	5.44	4.34	4.19	4.12	3.67	4.28	3.62, 4.01	
			99.6	78.6	80.0	66.6	73.4	69.3	73.3	
**E** →7)-l-*glycero*-α-d-*manno*-Hep*p*-(1→	*	*	4.98	4.05	3.95	3.93	3.72	4.23	3.69, 4.88	
			103.2	71.1	72.3	71.4	73.4	69.1	72.0	
**F** →4)-α-d-Gal*p*A-(1→	*	*	5.12	4.05	4.16	4.62	4.41			
			99.8	69.0	69.9	77.9	70.9	176.7		
**G** α-d-Glc*p*N-(1→	*	*	5.33	3.34	4.02	3.60	4.19	3.91^a^		
			95.6	55.1	70.4	76.6	73.2	60.0		
**H** α-d-Glc*p*-(1→	*	*	4.63	3.40	3.56	3.33	3.60	3.69, 3.98		
			103.2	74.5	76.3	71.6	76.5	62.7		
**I** →4)-α-d-Gal*p*A-(1→	*	*	5.47	3.93	4.26	4.48	4.67	-		
			102.5	69.6	72.2	80.9	72.5	175.6		
**J** α-d-Glc*p*N-(1→	*		5.29	3.39	3.99	3.63	4.27	3.69, 3.97		
			97.0	55.1	70.5	70.5	72.8	62.5		
**K** α-d-Glc*p*NAc-(1→		*	5.06	4.02	3.83	3.63	4.28	3.90^a^		2.13
			99.8	54.6	71.6	70.2	73.2	61.0		23.2
**L** Gly	*	*		3.90, 4.09						
			168.0	41.8						
**M** →β-d-Gal*p*-(1→		*	4.45	3.61	3.73	3.93	3.59	3.74, 3.80		
			103.6	71.0	72.7	71.4	75.1	63.2		

*ax*: Axial position; eq: Equatorial position; nd: Not resolved.

**Table 2:** Characteristics of the proteins encoded from *E. tarda* EIB 202 *waa*.

Protein	Homologus Protein	Predicted Function	% Identity/Similarity
ETAE_0083	HldE *Enterobacteriaceae*	ADP-l-*glycero*-d-*manno* Heptose-6-epimerase	85/91
ETAE_0082	WaaF *Enterobacteriaceae*	ADP-heptose:LPS heptosyl transferase II	76/86
ETAE_0081	WaaC *Enterobacteriaceae*	ADP-heptose:LPS heptosyl transferase I	78/84
ETAE_0080	WaaL *Klebsiella pneumoniae*	*O*-antigen ligase	29/46
ETAE_0079	Similar only to WabK *Klebsiella pneumoniae*	unknown	34/53
ETAE_0078	WapC *Plesiomonas shigelloides*	UDP-galacturonic a transferase α(1→7) to HepIII acid	76/89
ETAE_0077	WapB *Plesiomonas shigelloides*	UDP-*N*-acetyl glucosamine α(1→4) to GalAII	61/82
ETAE_0076	WabN *Klebsiella pneumoniae*	Protein deacetilase	77/89
ETAE_0075	WaaQ *Klebsiella pneumoniae*	ADP-heptose: LPS heptosyl	72/83
ETAE_0074	WabG *Klebsiella pneumoniae*	UDP-galacturonic acid transferase α(1→3) to HepIII	78/87
ETAE_0073	WabH *Klebsiella pneumoniae*	UDP-*N*-acetyl glucosamine transferase α(1→4) to GalAI	70/83
ETAE_0072	WaaA *Enterobacteriaceae*	3-deoxy-d-*manno*-octulosonic acid transferase	88/96
ETAE_0071	WapE *Plesiomonas shigelloides*	UDP-galactose transferase β(1→4) to l-HepI	82/97
ETAE_0070	CoaD *Enterobacteriaceae*	Phosphopantetehine adenylyltransferase	81/89
